# Submasseteric Tuberculous Lesion of Mandible: Report of a Case and Review of the Literature

**DOI:** 10.1155/2014/791630

**Published:** 2014-06-22

**Authors:** Donepudi Nanda Kishore, N. T. Geetha, K. V. Umashankara, Kirthi Kumar Rai

**Affiliations:** Department of Oral, Maxillofacial and Reconstructive Surgery, Bapuji Dental College and Hospital, Davangere, Karnataka 577004, India

## Abstract

Tuberculosis is still a major health hazard in the developing world, while its incidence has recently started to escalate after decreasing for many years. It is a chronic granulomatous disease that can affect any part of the body, including the oral cavity. Oral lesions of tuberculosis, though uncommon, are seen in both the primary and secondary stages of the disease. This paper presents a case of tuberculosis of the submasseteric space, manifesting as a persistent swelling at ramus and angle of mandible. The diagnosis was confirmed based on histopathology after an open incisional biopsy. Patient underwent antituberculosis therapy and his extraoral swelling completely resolved after 4 months of the therapy. The purpose of this paper is to emphasize the importance of early and definitive diagnosis of orofacial tuberculosis, to recognize it based on signs and symptoms, and to refer the patients suspected of active tuberculous infection for appropriate medical treatment.

## 1. Introduction

Extrapulmonary tuberculosis (TB) is an uncommon form of chronic infection occurring rarely in the head and neck region, seen in only 1% of patients, excluding tuberculous lymphadenitis of neck [1]. The diagnosis of this paucibacillary lesion is often overlooked because of its variable mode of insidious presentation with no specific pathognomic signs [2]. Despite rare occurrence, the differential diagnosis of this tuberculous granulomatous lesion must always lurk in the dental clinicians mind, as this spreads by way of airborne droplets. An early diagnosis with prompt treatment can prevent complications and potential contaminations. Here, we report a case of tuberculous lesion at submasseteric space of mandible, which resolved completely after antituberculosis therapy.

## 2. A Case Report

**Figure 1:**
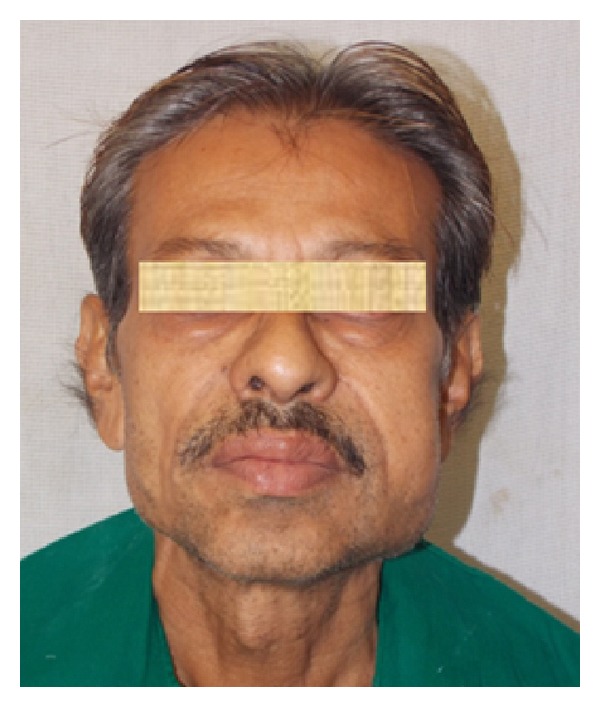
Preoperative picture showing an extraoral swelling at the left angle and ramus region of the mandible.

**Figure 2:**
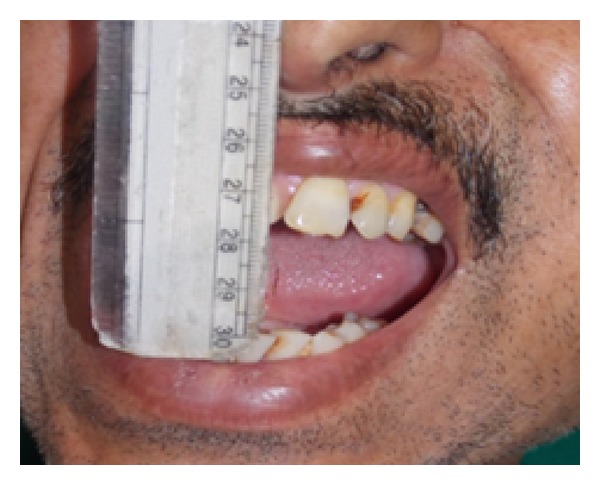
Preoperative picture shows mild trismus with MO of 23 mm.

**Figure 3:**
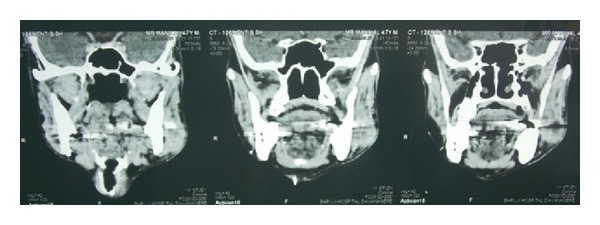
CT scan reveals cystic lesion in masseteric space involving the ramus of the left mandible with lytic and sclerotic area.

A 48-year-old man was reported to the Department of Oral and Maxillofacial Surgery, with a mild swelling on the left side of the cheek. On physical examination, he was afebrile, moderately built, and nourished with no distress. Extraoral examination revealed a mild swelling at the angle of mandible associated with slight tenderness on palpation. Intraoral examination revealed left buccal vestibular obliteration at retromolar region and grossly decayed 38, which was tender on percussion. The patient was started on a 5-day course of antibiotics and, during this course, atraumatic extraction of grossly decayed 38 was done under LA. The extracted socket healed well in time uneventfully. Patient returned back after 2 months with a gradual increase in extraoral swelling (Figure 1) and mild trismus with mouth opening of 23 mm (Figure 2), despite the postextraction 5-day course of antibiotics. On careful examination, a solitary swelling with moderately defined borders from the midbody of mandible up to angle and ramus region was seen. On clinical palpation, the mass was firm, nonpulsatile, and nonfluctuant with normal overlying skin and was fixed to underlying structures, measuring 2 × 3 cm. Mild tenderness could be elicited on palpation. CT scan (Figure 3) revealed cystic lesion in masseteric space involving the ramus of the left mandible with lytic and sclerotic area. Thus, an impression of osteomyelitis with submasseteric abscess was suggested.

**Figure 4:**
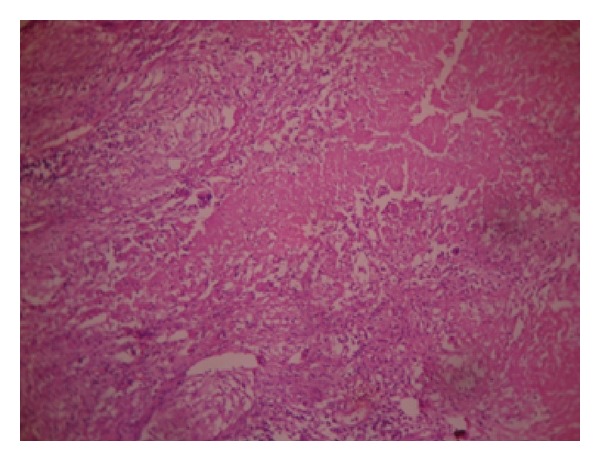
Microscopic picture shows features of caseating granulomatous inflammation.

The routine blood investigation was found to be normal except slight rise in ESR to 40 mm/hr. HIV and HBsAg were also found to be negative (nonreactive). An intraoral open incisional biopsy was performed under general anesthesia. Two bits of specimen were cut from the region of masseter and parotid and sent for histopathological examination. Later, it was confirmed as granulomatous tuberculosis. Microscopic picture (Figure 4) shows features of caseating granulomatous inflammation.

**Figure 5:**
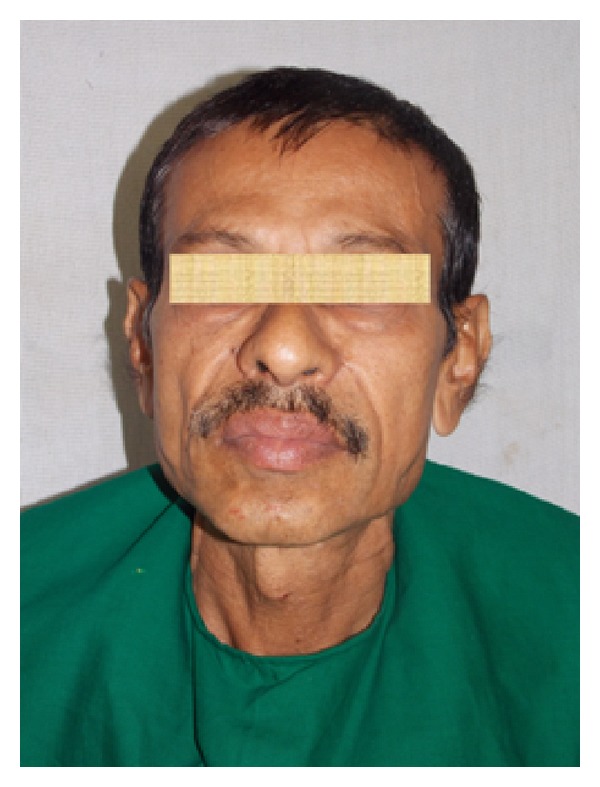
Picture after 4 months of antituberculous therapy, with completely resolved extraoral swelling.

On enquiry, the patient revealed no history of TB, but he had history of cough, cold, recurrent pneumonia, and slight weight loss one year ago. The patient was asked for all his documents of previous reports and treatment. AFB sputum was negative at that time. CT thorax revealed normal hili with no Koch's positive but with slight right basal consolidation, for which he was treated with tab Azithromycin 500 mg, tab Montreal (Leukotriene receptor antagonist), and tab Sinarest (Cetrizine) for 12 days. With this past history and histopathological report of incisional biopsy, he was referred to National TB Control Programme Center for further medical management. The patient was given a regimen of Rifampicin 600 mg/day, Ethambutol 2tab 600 mg, Isoniazide 2tab 300 mg, and 2tab Pyrazinamide thrice a week for 1 month. Later, the doses of these antitubercular drugs were reduced for another 3 months. The response to antituberculosis therapy was excellent and the extraoral swelling completely resolved with only a peanut-sized fibrosis (Figure 5) by the end of the 4th month of the therapy.

## 3. Discussion

Tuberculosis is still a major health hazard in the developing world, while its incidence has recently started to escalate after decreasing for many years. Primary infection of orofacial tissues does occur without systemic infection, but it is extremely rare and generally occurs in younger patients [3]. The target organ of mycobacterium tuberculosis is the bronchopulmonary apparatus and the head and neck are usually secondary. Primary involvement is more common in children and adolescents than in adults [4, 5]. Primary orofacial tuberculosis usually involves the gingival, mucobuccal folds and inflammatory foci adjacent to the teeth or extraction sites [6]. Secondary oral TB can occur in all age groups but is most common in middle and older age groups. It occurs from a healed primary focus or due to endogenous spread of the infection. Secondary TB is usually chronic in nature and can cause considerable destruction of the involved tissue with caseation, cavity formation, and fibrosis [7].

The integrity of the oral epithelium and the inhibitory effect of saliva cause relative resistance to infection by* M. tuberculosis* bacilli [8]. Orofacial lesions may appear in various forms as ulcers, nodules, fissures, tuberculomas, or granulomas [9]. These lesions may be single or multiple, painful or painless, and usually appear as irregular, well-circumscribed ulcer. However, they can begin as nodules, fissures, or vesicles and then slowly increase in size when they become chronic [9]. Here in our case, the lesion was present at the angle and ramus region of the mandible. The patient had fair to healthy oral hygiene with no source of odontogenic infection or breach in oral mucosa. The extraction socket had healed well in time. There was no evidence of direct inoculation of AFB. There were no supportive clinical findings of swelling that could be linked to an odontogenic cause, cyst, or neoplasm. The firm mass with moderately defined borders, which is fixed to the underlying structures, could be suspicious of either a fibroosseous lesion or an ameloblastic fibroma. There are undoubtedly patients in whom the proper diagnosis and therapy are delayed or missed entirely. Misdiagnosis can sometimes lead to surgical excision of the entire fibrosed or calcified mass, which often resolves completely by antituberculosis therapy only. The spread to the angle and ramus region of mandible in this case can be explained only by arterial supply of masseter and medial pterygoid muscles, thus supporting theory of spread by a hematogenous route. The destruction of bone at angle region appears to be from outside to inside in contrast to normal pattern in osteomyelitis owing to odontogenic infection. The masseter involvement adjacent to bone shows that infection progressed from the attached muscle to the angle of mandible. USG certainly has some role in soft tissue masses, abscesses, and the degree and extent of tendon and tendon sheath involvement. CT scan is helpful for the detection of osseous or joint involvement, the presence or absence of periosteal reaction and soft tissue calcifications, sclerosis, and soft tissue abscesses. USG and CT scan are particularly useful for guiding fine needle aspiration or biopsy to provide material for histopathological examination.

## 4. Transmission of Tuberculosis

It spreads by way of airborne droplets by cough, sneeze, or talk generating up to 3000 droplet nuclei of varying size. Smaller droplets harbor several bacilli which can travel to the terminal bronchioles and the alveoli settle, exposing the mycobacteria to host tissues [6]. These, in turn, get engulfed by pulmonary macrophages, which migrate to the regional lymphatic circulation [6]. Here, they may take up residence within nearby lymph nodes or spread throughout the body [6]. Bacilli may also invade blood vessels, with subsequent systemic distribution [10]. The interaction between the mycobacteria and the host inflammatory cells creates a focal zone of necrosis that over time may undergo fibrosis and calcification [10]. People with extrapulmonary tuberculosis without respiratory or oral signs or symptoms are not at risk for spreading the disease [11]. Only the persons with active disease are infectious to others. An active form can be suspected when medical history includes cough, production of sputum and blood (hemoptysis), and chest pain [12]. Other nonspecific symptoms, such as anorexia, fatigue, weight loss, fever, and night sweats, are often associated with active TB. Patient who is diagnosed with TB has to be questioned regarding status of disease. It is vital to make a distinction between asymptomatic infection, inactive disease, and active disease.

## 5. Conclusion

To conclude, tuberculosis of the orofacial region is relatively rare and has largely become a forgotten diagnosis of orofacial region. Clinicians are not sensitized to the disease as part of differential diagnosis. Any chronic infection in the maxillofacial region should be ruled out for TB. Surgeons might sometimes attempt for surgical excision of entire fibrosed or calcified mass, which can cause surgical morbidity to the patient. It is always fair enough to do an incisional biopsy, when in doubt about exact clinical and radiological diagnosis. The final diagnosis can be established only by histopathological confirmation and microbiological study of tissue specimen for a definitive diagnosis and appropriate treatment of tuberculosis.
